# InclusiveHAR: A smartphone-based dataset for human activity recognition across diverse physical abilities

**DOI:** 10.1016/j.dib.2026.112620

**Published:** 2026-02-28

**Authors:** Seyed Reza Kamel Tabbakh, Iman Naeimi, Kosar Naghavi, Fatemeh Majidi Nasab, Mehran Ghaffarian, Vahideh Nobahari, Mahdieh Sadat Sharifi Moghaddam Kakhki, Hojjat Farrahi Farimani, Sanaz Rouhparvar

**Affiliations:** a Department of Computer Engineering, Ma.C., Islamic Azad University, Mashhad, Iran; b Department of Computer Engineering, Amirkabir University of Technology, Tehran, Iran

**Keywords:** Human activity recognition, HAR, Wearable sensors, Smartphone, Individuals with disabilities

## Abstract

Human Activity Recognition (HAR) has numerous applications in healthcare, rehabilitation, athletics, and smart environments. Effective AI models rely on diverse and representative datasets to achieve robust generalization. However, the majority of existing HAR datasets are collected exclusively from non-disabled individuals, limiting their applicability in real-world healthcare scenarios involving the elderly or individuals with disabilities. To address this limitation, we introduce InclusiveHAR, a novel smartphone-based HAR dataset collected from 20 participants, including 10 non-disabled individuals and 10 individuals with disabilities, of whom five had a single disability and five had multiple distinct conditions. Participants performed six daily activities: walking, standing, sitting, jogging, ramp ascent, and ramp descent. The dataset captures a wide range of movement patterns and behavioural variability, with particular emphasis on differences in activity execution observed in individuals with disabilities. Data were collected using an iPhone 14 Pro at a sampling rate of 50 Hz (one sample every 20 ms). The SensorLog app was used to lock the rate at 50 Hz. To illustrate the potential use of the dataset, a baseline evaluation is provided under multiple training scenarios using the MLP machine learning model. In this paper, we report and evaluate the performance of dataset against K-NN, SVM, and XGBoost models. In addition, the dataset is accompanied by detailed feature descriptions and comprehensive documentation of the data collection protocol, enabling transparent analysis, reproducibility, and future comparative studies. The InclusiveHAR dataset offers a valuable resource for investigating activity recognition performance across diverse participant groups and for supporting the development of inclusive HAR systems in healthcare and assistive technology applications.

Specifications Table

**Table:** 

Subject	Computer Sciences
Specific subject area	Human Activity Recognition (HAR) for individuals with disabilities
Type of data	Table (.csv)
Data collection	Data were collected using the internal sensors of an iPhone 14 Pro (accelerometer, gyroscope, magnetometer, motion, and GPS sensors) at a sampling rate of 50 Hz. The SensorLog app was used to lock the rate at 50 Hz. The phone was placed vertically in a waist pouch worn by participants.
Data source location	Tavan-Yaban Educational and Rehabilitation Center for People with Disabilities, a non-governmental organization (NGO) in Mashhad, Iran
Data accessibility	Repository name: Mendeley Data DOI link: 10.17632/r78dn3f6nc.4 License: CC BY 4.0 (Creative Commons Attribution 4.0 International) Direct URL to dataset: https://data.mendeley.com/datasets/r78dn3f6nc/4
Related research article	None

## 1 Value of the Data

• To the best of our current knowledge, InclusiveHAR is the first publicly accessible HAR dataset that fills a major gap in publicly available resources for human activity recognition (HAR), particularly in studies involving individuals with disabilities, where labeled movement data remain scarce. This dataset offers a foundational step toward more inclusive research.
• It contains sensor measurements from 20 participants—both individuals with disabilities and non-disabled— performing six common daily activities using standard smartphone sensors.
• HAR plays an essential role in healthcare applications for older adults and individuals with disabilities. This dataset provides a reliable and valuable resource for advancing HAR systems in various domains. These include healthcare and elderly-care solutions, fall-warning systems and emergency-need detection systems, rehabilitation analysis, assistive device and smart- wheelchair development, patient monitoring, and the creation of personalized activity models, ultimately improving detection accuracy in real-world settings.
• The dataset supports researchers in machine learning and pattern recognition by enabling the development of models capable of distinguishing activity patterns between individuals with disabilities and non-disabled individuals with greater precision.
• The dataset includes demographic details—such as gender, age, height, weight, and disability status—which highlight the importance of representation and diversity in building inclusive HAR systems.
• The dataset enables comparative evaluation of HAR models across individuals with disabilities and non-disabled participants, analysis of movement variability across different disability types, investigation of the impact of assistive devices on sensor signals, and the development of personalized or subject-adaptive activity recognition models for healthcare applications.

## 2 Background

In recent years, HAR has gained significant attention due to its wide range of applications in healthcare, elderly care, sports, and smart environments [[1], [2], [3]]. Among various sensing approaches, wearable and smartphone-based sensors have become the most popular tools for collecting human motion data because of their portability and accessibility [4,5]. Machine learning models are increasingly used to analyse these signals and recognize daily activities, relying heavily on the availability of large and diverse data for effective training and generalization [5]. Despite extensive research in HAR, most publicly available datasets have been collected from non-disabled individuals, which limits their applicability in healthcare scenarios involving elderly people or those with physical disabilities [[6], [7], [8]]; according to the World Health Organization (WHO), approximately 16 % of the world population lives with some form of disability [6]. Datasets that capture the movement characteristics of these individuals remain scarce. To address this gap, the InclusiveHAR dataset was developed using smartphone sensors, including an accelerometer, gyroscope, magnetometer, and GPS, to record six daily activities performed by both individuals with disabilities and non-disabled participants. This dataset provides a valuable foundation for developing inclusive and adaptive HAR models capable of recognizing diverse movement patterns in real-world healthcare and rehabilitation applications.

## 3 Data Description

The dataset presented in this study was collected at a rehabilitation centre in Mashhad, Iran. It consists of raw multimodal smartphone sensor data recorded from 20 adult participants performing six daily activities: walking, standing, sitting, jogging, ramp ascent, and ramp descent. Participants were divided into two equal groups: ten non-disabled individuals and ten individuals with Several types of disabilities, including cerebral palsy, multiple sclerosis, and congenital or genetic disorders.

**Fig. 1:**
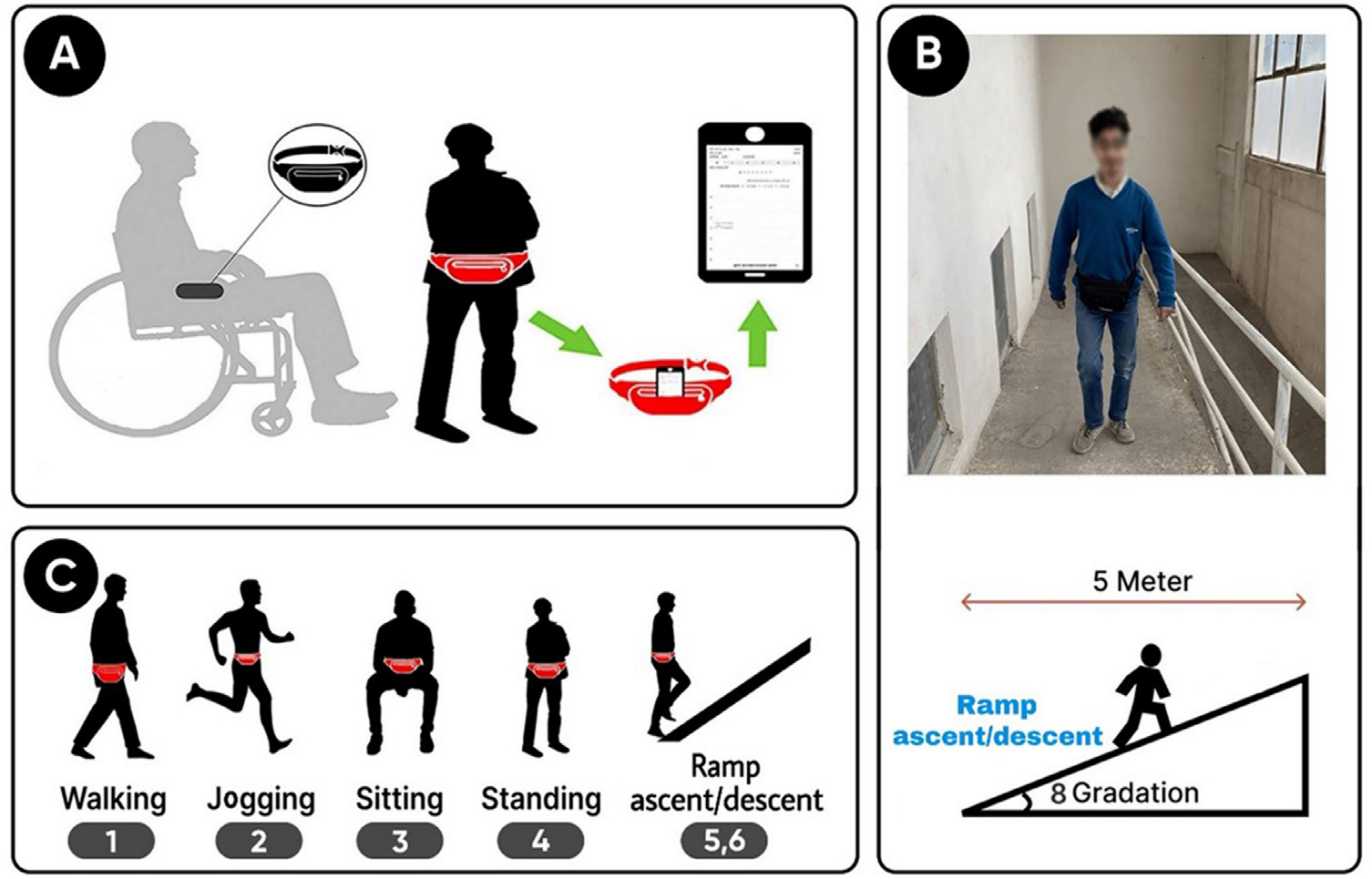
(a) Placement of the smartphone inside the waist pouch during data recording. (b) The ramp used at the rehabilitation center for wheelchair activity data collection. (c) Six activities.

Data were captured using an iPhone 14 Pro smartphone placed vertically in a waist pouch worn by each participant (Fig. 1(a)). The smartphone’s internal sensors continuously recorded motion and location data at a sampling rate of 50 Hz (one sample every 20 ms). The SensorLog app was used to lock the rate at 50 Hz. Each activity was performed for 60 s and repeated three times to ensure reliable data collection. To improve data quality, the first and last 50 samples of each recording were removed to eliminate transient noise and start/stop motion artifacts. The entire data collection process was supervised by professional rehabilitation experts to ensure participant safety and data reliability (Fig. 1(b)). Activities involving ascending and descending movements were physically conducted on an inclined ramp with an 8 % slope to ensure participant safety; however, these activities are labeled as “ramp ascent” and “ramp descent” to reflect the functional movement patterns rather than the physical environment. Additionally, ``walking'' labels for wheelchair users refer to manual propulsion.

The final dataset contains 396,603 time-stamped samples organized in a single CSV file. Each row represents one time-step observation and includes 30 sensor-based feature columns derived from location, accelerometer, gyroscope, magnetometer, and motion sensors. Table 2 presents sample rows illustrating the structure and content of the dataset. The features are grouped into the following categories:

Table 1 provides a comprehensive summary of the raw sensor features available in the InclusiveHAR dataset. All signals are provided in raw form without any segmentation or windowing, allowing researchers to apply customized preprocessing, segmentation, and feature extraction pipelines. The magnetometer features (X, Y, Z) correspond to raw sensor readings, while the motion-based magnetic field variables represent calibrated and sensor-fused measurements provided by the device motion framework. This distinction avoids confusion regarding redundancy and ensures clarity in feature description.

**Table 1:** Summary of features included in the InclusiveHAR dataset.

Feature Name	Sensor / Source	Axes	Description
id	System	–	Unique identifier of the data sample
location	GPS	Latitude / Longitude / Altitude / Speed / Course / VerticalAccuracy	GPS-based location, speed, and vertical accuracy measurements
accelerometer	Accelerometer	X / Y / Z	Linear acceleration along three axes
gyroscope	Gyroscope	X / Y / Z	Angular velocity around three axes
magnetometer	Magnetometer	X / Y / Z	Magnetic field strength along three axes
motionOrientation	Motion Sensor	Yaw / Roll / Pitch	Device orientation angles
motionRotationRate	Motion Sensor	X / Y / Z	Rotation rate around three axes
motionUserAcceleration	Motion Sensor	X / Y / Z	User-generated acceleration along three axes
motionGravity	Motion Sensor	X / Y / Z	Gravity vector along three axes
motionMagneticField	Motion Sensor	X / Y / Z	Magnetic field vector along three axes
Label	Annotation	–	Ground-truth activity label (e.g., walking, standing, jogging)

**Table 2:** Example rows of raw sensor data illustrating the format and organization of the dataset.

feature	accelerometer AccelerationX	accelerometer AccelerationY	accelerometer AccelerationZ	gyroRotationX	gyroRotationY	gyroRotationZ
1	0.934189	−0.08328	0.498001	−0.21535	−0.04921	0.599987
2	0.91893	0.136505	0.493591	−0.21535	−0.04921	0.599987
3	0.930634	0.103027	0.428772	0.00981	0.097381	−0.09921
4	0.98671	0.036652	0.461288	−0.08679	0.019043	−0.15612
5	0.908203	0.06279	0.402557	−0.09093	0.051089	−0.17055
6	0.845886	0.080872	0.453583	−0.1331	0.180335	−0.09486
7	0.851563	0.095795	0.537491	−0.1568	−0.13976	−0.04543

In addition to activity labels, the dataset includes a binary indicator specifying disability status, enabling direct comparison of model behavior between individuals with disabilities and non-disabled participants using the same recognition framework. Furthermore, metadata such as subject identifiers, demographic information, and assistive device usage allow researchers to explore subject-adaptive and personalized HAR approaches, as well as the influence of assistive devices on recorded motion patterns.

## 4 Experimental Design, Materials, and Methods

This section describes the methodology used to collect, organize, and validate the InclusiveHAR dataset. The dataset was specifically developed to address a major limitation in existing HAR research—namely, the underrepresentation of individuals with disabilities in training data.

Unlike conventional datasets that primarily include non-disabled participants, the InclusiveHAR dataset incorporates activity patterns from both non-disabled individuals and individuals with physical disabilities, allowing for more inclusive and generalizable HAR model training.

### 4.1 Data collection

The dataset was collected at a rehabilitation center in Mashhad, Iran, using an iPhone 14 Pro smartphone equipped with built-in motion and location sensors. The device recorded data at a sampling frequency of 50 Hz. The SensorLog app was used to lock the rate at 50 Hz. During data collection, the phone was placed vertically inside a waist pouch, secured around each participant’s waist, as shown in Fig. 1(a).

To ensure high data quality, the signals were sampled at 50 Hz (one sample every 20 ms). The SensorLog app was used to lock the rate at 50 Hz. Each activity lasted 60 s. Both non-disabled and individuals with disabilities performed the activities of ascending and descending stairs (Fig. 1(c)); however, for safety reasons, all stair-related activities were conducted on a ramp with an 8 % incline (Fig. 1(b)). This design allowed consistent comparison across participants while preventing potential injuries for those with mobility limitations (Fig. 1(b)). The diversity of disability types and mobility aids introduces distinct movement patterns, providing opportunities for future analysis of activity recognition across different impairment categories.

The released dataset consists of raw, continuous sensor recordings. For experimental analysis, researchers may optionally segment the raw signals into fixed-length sliding windows, such as 2-second windows with a 50 % overlap, depending on the target application and model architecture. This segmentation is not imposed on the dataset and is provided only as a recommended preprocessing strategy. Throughout the data acquisition process, particular attention was given to participant safety, comfort, and privacy. All participants provided informed consent before participating in the study.

### 4.2 Subjects and activities

This dataset involves data from 20 participants who were divided into two groups: non-disabled individuals and individuals with disabilities. Table 1 presents the participants’ information, types of disabilities, and the assistive devices used. The first 10 rows of the table represent the non-disabled individuals, and the last 10 rows belong to the individuals with disabilities. The non-disabled group consists of 10 participants, including 5 men and 5 women, with an age range of 22 to 25 years, an average height of 171.7 cm, and an average weight of 73.6 kg. All activities for this group were conducted under the same conditions. The group with disabilities consists of 10 participants, including 8 men and 2 women, with an age range of 24 to 48 years, an average height of 167.2 cm, and an average weight of 66.2 kg. This group includes participants with Several types of disabilities, such as cerebral palsy (subjects 15, 16, and 19), multiple sclerosis (subject 13), and congenital or genetic disorders (subject 18). The activity conditions for each participant with disabilities were adjusted based on their specific abilities.

This dataset includes six common daily human activities: walking, standing, sitting, jogging, ramp ascent, and ramp descent. These activities were selected due to their frequency in daily life. Each activity was performed for 60 s and repeated three times to collect sufficient data for accurate movement pattern analysis.

Some participants with disabilities used assistive devices such as canes, walkers, or wheelchairs during data collection. The resulting variations in sensor signals were intentionally preserved, as they reflect realistic movement conditions in rehabilitation and healthcare environments and enable future studies on the impact of assistive devices on HAR performance (Table 3).

**Table 3:** Characteristics of non-disabled subjects and individuals with disabilities.

User ID	Gender	Age	Height (cm)	Weight (kg)	Disability	Assistive Device
1	M	25	179	69	None	None
2	M	24	172	99	None	None
3	M	24	190	71	None	None
4	M	24	185	93	None	None
5	M	22	180	91	None	None
6	F	24	161	63	None	None
7	F	23	157	70	None	None
8	F	24	162	54	None	None
9	F	22	162	62	None	None
10	F	22	169	64	None	None
11	M	35	170	45	Muscle Spasm	Walker
12	M	48	174	80	Polio (Difficulty Walking)	None
13	M	35	182	82	MS, Strabismus	None
14	M	24	175	50	Muscle Spasm	None
15	M	25	172	90	Cerebral Palsy (Severe CP)	None
16	M	25	164	53	Cerebral Palsy (Difficulty Walking)	None
17	M	43	174	69	Ataxia	Cane
18	M	34	150	60	Congenital-Genetic	Wheelchair
19	F	29	155	60	Cerebral Palsy	Wheelchair
20	F	43	160	113	Hip Dislocation (Difficulty Walking)	None

### 4.3 Performance comparison using a baseline MLP model

Baseline experimental evaluation was conducted using a Multilayer Perceptron (MLP) classifier as a reference model. The MLP was selected due to its widespread use in human activity recognition studies and its capability to model non-linear relationships in sensor-based time-series data [9].

All experiments were implemented using Python (version 3.10.12) and the scikit-learn library. After preprocessing, the raw sensor signals were divided into training and testing sets, with 80 % of the data allocated for training and 20 % for testing [9]. The dataset was evaluated under different training scenarios to examine variations in model performance when trained on datasets with different participant group compositions. These scenarios included training exclusively on data from non-disabled individuals and training on a combined dataset comprising both non-disabled and individuals with disabilities.

The MLP hyperparameter values were empirically selected and adjusted to ensure stable model convergence. The final model configuration consisted of a single hidden layer with 100 neurons and a maximum of 300 training iterations. This configuration is reported solely to support reproducibility, and no claim is made regarding its optimality. Model performance was assessed using standard classification metrics, including accuracy, precision, recall, and F1-score [1,9]. The quantitative results obtained under the considered training scenarios are summarized in Table 4.

**Table 4:** Performance comparison of the MLP model under different training scenarios.

	Accuracy (%)	Precision (%)	Recall (%)	F1-score (%)
Scenario 1 (non-disabled data)	12.87	12.08	12.87	12.15
Scenario 2 (Both individuals with disabilities and non-disabled data)	42.54	42.4	42.54	39.37

As shown in Table 4, the baseline MLP model exhibits noticeable performance differences across training scenarios. When the model is trained exclusively on data from non-disabled individuals, its ability to recognize activities performed by individuals with disabilities is limited, which may be attributed to differences in movement patterns and activity execution between the two groups. In contrast, training the model on a combined dataset that includes data from individuals with disabilities leads to improved performance across all evaluation metrics. These observations highlight the importance of diverse and representative training data and demonstrate the suitability of the InclusiveHAR dataset for analyzing the impact of participant composition on human activity recognition performance.

## Limitations

One limitation of this dataset is the relatively small number of participants, which includes 20 individuals (10 non-disabled and 10 with disabilities). While the dataset provides valuable insights into the activity patterns of individuals with mobility impairments, the limited participant pool restricts the generalizability of the findings to a broader population. Additionally, the disability group includes only a few categories of disorders (such as cerebral palsy, multiple sclerosis, and congenital or genetic conditions), which may not fully represent the full spectrum of physical disabilities.

Future data collection should include a larger and more diverse participant group and a wider range of disability types to improve model robustness and applicability in real-world healthcare and assistive environments.

## Ethics Statement

Before the experiment, the experimental conditions were explained to all participants, and each participant signed a written informed consent form prior to participation. The study was approved by the Ethics Committee of the Faculty of Medicine, Islamic Azad University, Mashhad Medical Sciences Unit, and was assigned the ethical approval code IR.IAU.MSHD.REC.1404.170. The study was conducted in accordance with the Declaration of Helsinki.

## CRediT Author Statement

**Seyed Reza Kamel Tabbakh:** Conceptualization, Investigation, Resources, Writing – review and editing, Supervision, Project administration; **Iman Naeimi:** Conceptualization, Investigation, Resources, Data curation; **Kosar Naghavi:** Methodology, Validation, Resources, Data curation, Writing – review & editing, Project administration; **Fatemeh Majidi Nasab:** Resources, Data curation, Writing – review and editing; **Mehran Ghaffarian:** Methodology, Validation, Writing – review and editing; **Vahideh Nobahari:** Resources, Writing – review & editing; **Mahdieh Sadat Sharifi Moghaddam Kakhki:** Resources, Data curation, Writing – review & editing; **Hojjat Farrahi Farimani:** Resources, Writing – review & editing; **Sanaz Rouhparvar:** Resources, Writing – review & editing.

## Data Availability

Mendeley DataInclusiveHAR: A Smartphone-Based Dataset for Human Activity Recognition Across Diverse Physical Abilities https://data.mendeley.com/datasets/r78dn3f6nc/4.
